# A pangenome‐based approach reveals genes associated with polyploidy and apomixis in *Eragrostis curvula*

**DOI:** 10.1002/tpg2.70303

**Published:** 2026-09-21

**Authors:** Giacomo Bongiorno, José Carballo, Juan Pablo Selva, Diego Zappacosta, Emidio Albertini, Viviana Echenique

**Affiliations:** ^1^ Department of Agricultural, Food and Environmental Sciences University of Perugia Perugia Italy; ^2^ Centro de Recursos Naturales Renovables de la Zona Semiárida Bahía Blanca Argentina; ^3^ Departamento de Agronomía Universidad Nacional del Sur Bahía Blanca Argentina

## Abstract

*Eragrostis curvula* is a forage grass species in which multiple cytotypes coexist and shifts between sexual and apomictic reproduction are common. This study investigates genetic variation among these cytotypes in relation to ploidy levels and reproductive modes. To this end, a panel of accessions was assembled to capture the species’ intraspecific diversity. The panel was used to construct a whole‐genome sequencing‐based linear pangenome by iteratively mapping Illumina short reads and de novo assembling unmapped reads, thereby expanding the available reference genome and improving the representation of the species’ gene pool. The panel and the resulting pangenome were then used for gene content detection, functional annotation, and variant calling. Using this approach, extensive variation in gene content was observed among *E. curvula* accessions, with polyploid cytotypes containing a higher fraction of newly assembled pangenome sequences than diploids. In addition, candidate genes associated with polyploidization and apomixis were identified. Polyploid‐associated genes were mainly associated with cell‐cycle regulation and DNA replication, whereas apomixis‐associated genes were mainly associated with post‐transcriptional regulation. Moreover, phylogenetic relationships among cytotypes were inferred, and allele‐frequency distributions revealed ploidy‐associated differences among accessions. Thus, this work identified candidate genes associated with polyploidy and apomictic reproduction, highlighting ploidy‐associated changes in gene content and post‐transcriptional regulatory processes and providing insights into how polyploidization and reproductive shifts have shaped genome evolution in this agamic grass species.

## Core Ideas

- We built the first *Eragrostis curvula* pangenome across a panel of cytotypes, expanding the species’ reference gene pool.
- Cytotypes with different ploidy levels and reproductive modes exhibit extensive gene content variation.
- Gene groups associated with polyploidy and apomixis were identified through gene presence/absence variation analysis.
- Single‐nucleotide polymorphism (SNP) markers enabled inference of phylogenetic relationships among cytotypes.

## Plain Language Summary

Plants can differ not only in chromosome number but also in reproductive strategy. Some plants reproduce sexually, while others reproduce asexually through a mechanism called apomixis, producing genetically identical offspring without fertilization. Many apomictic plants are also polyploid, with extra sets of chromosomes, but how these traits are linked remains unclear. *Eragrostis curvula* (weeping lovegrass) is a model species, as it includes cytotypes with different reproductive modes and ploidy levels. We developed a pangenome (i.e., a genome assembly built from multiple individuals), enabling the identification of gene presence/absence variations (PAVs). By analyzing PAVs, we observed that polyploid plants generally contain more genes. Our results suggest that increased chromosome number and asexual seed formation co‐occur due to two distinct groups of candidate genes, associated with polyploidy and apomixis, respectively.

**Abbreviations**
AF: allele frequency
BAM: Binary Alignment Map
BUSCO: Benchmarking Universal Single‐Copy Orthologs
cDNA: complementary DNA
GO: Gene Ontology
GVCF: Genomic Variant Call Format
InDel: insertion/deletion
miRNA: microRNA
mRNA: messenger RNA
PAV: presence/absence variation
PCA: principal component analysis
RT‐PCR: reverse transcription polymerase chain reaction
SNP: single‐nucleotide polymorphism
TF: transcription factor
TR: transcriptional regulator
TRAF: tumor necrosis factor receptor‐associated factors
VCF: Variant Call Format
WGS: whole‐genome sequencing

## 1 INTRODUCTION

Before the adoption of pangenomic approaches, plant genomic studies relied on single reference genomes. The first complete genome of *Arabidopsis thaliana* (The Arabidopsis Genome Initiative, 2000), followed by those of important crops such as *Oryza sativa* (J. Yu et al., 2002), *Vitis vinifera* (The French–Italian Public Consortium for Grapevine Genome Characterization, 2007), *Sorghum bicolor* (Paterson et al., 2009), *Zea mays* (Schnable et al., 2009), and *Glycine max* (Schmutz et al., 2010), marked a period of rapid growth in plant genome sequencing. This expansion has been driven by the advent of next‐generation sequencing technologies, which have revolutionized genomics by making complete genome sequencing accessible to many laboratories (Van Dijk et al., 2014). As a result, more than 4500 genomes from over 1400 plant species have been sequenced to date (Bernal‐Gallardo & de Folter, 2024). Sequencing efforts focused primarily on model species, major crops, and related wild species (M. W. Li et al., 2025), aiming to produce at least one reference genome for each species. However, as new genome assemblies become increasingly available, it has become evident that a single reference genome is insufficient to capture the full diversity within a species (Golicz, Batley, et al., 2016). The pangenome concept, introduced in 2005, addressed this problem by distinguishing between core genes shared by all individuals and accessory or variable genes found only in some individuals. Originally proposed for prokaryotes (Tettelin et al., 2005), the concept was later extended to eukaryotes, including plants (Morgante et al., 2007). However, the large size, high ploidy, and repetitive nature of many plant genomes (Michael & VanBuren, 2015; Schatz et al., 2012) limited the adoption of pangenomic approaches until breakthroughs in sequencing technologies and bioinformatics made them widely applicable.

Pangenomes are reference assemblies built from genomic sequences of multiple individuals rather than a single individual (Golicz et al., 2020) and thus represent a type of reference that can reduce the bias inherent in using a single reference that does not capture the full variability of a species (W. Li et al., 2022; J. Wang et al., 2023). Currently, three main approaches are used to construct plant pangenomes: graph‐based assembly, de novo assembly and comparison, and iterative assembly (Bayer et al., 2020; Hu et al., 2024, 2025). In the iterative assembly approach, reads are aligned to a reference, and unmapped reads are assembled into non‐reference sequences, which are then integrated into a linear pangenome. This method has been used successfully to identify presence/absence variations (PAVs) in several plant species, including *Brassica oleracea* (Golicz, Bayer, et al., 2016), *Brassica napus* (Hurgobin et al., 2018), *Triticum aestivum* (Montenegro et al., 2017), *Cajanus cajan* (Zhao et al., 2020), and *Sorghum bicolor* (Ruperao et al., 2021). Therefore, the iterative mapping approach is a cost‐effective strategy for identifying genes across diverse species or populations that cannot be targeted by a single whole‐genome assembly.

*Eragrostis curvula* (Schrad.) Ness, commonly known as weeping lovegrass, is a perennial C4 plant in the Poaceae family that has been introduced worldwide for forage production and soil conservation (Voigt et al., 2004). It is well adapted to arid and semi‐arid regions because of its drought tolerance and ability to thrive in nutrient‐poor soils (Luciani et al., 2012). However, this species remains an orphan crop with limited domestication and selection and no long‐term breeding program (Carballo, Zappacosta, Selva, et al., 2021). Taxonomically, *E. curvula* belongs to the polyphyletic genus *Eragrostis*, which includes over 350 species (Watson & Dallwitz, 1992) and exhibits extensive morphological and genetic diversity (Zeid et al., 2011). At the species level, *E. curvula* presents several cytotypes that vary in ploidy and reproductive mode (Carballo, Zappacosta, Selva, et al., 2021). In nature, diploid (2x) cytotypes are always sexual, whereas most polyploids (4x–8x) reproduce through apomixis, a type of asexual reproduction that allows plants to produce clonal seeds (Voigt et al., 2004). The association between polyploidy and apomictic reproduction is widespread among angiosperms. Indeed, apomictic plants that produce unreduced parthenogenetic eggs are predominantly polyploid and have been reported in at least 33 of 460 angiosperm families (Carman, 1997).

In *E. curvula*, most polyploid cytotypes reproduce through facultative apomixis and can therefore produce both zygotic and clonal seeds within the same plant (Koltunow et al., 1995). However, in rarer cases, tetraploid cytotypes are either sexual or fully apomictic, producing exclusively zygotic or clonal seeds, respectively. Diplospory in *E. curvula* has been shown to co‐segregate with molecular markers located within a specific genomic region that was subsequently associated with a chromosome‐scale contig of the Victoria reference genome (Carballo, Zappacosta, Selva, et al., 2021). Moreover, *E. curvula* exhibits a distinctive type of embryo sac development known as the “*Eragrostis* type,” in which meiosis is suppressed, and embryo sac development involves only two mitotic divisions (Carballo, Zappacosta, Selva, et al., 2021; Crane, 2001). Cytogenetic studies have revealed both bivalent and multivalent chromosome pairing during meiosis in *E. curvula* (Poverene, 1988; Zeid et al., 2011). In cv. Tanganyika INTA, the high frequency of multivalent configurations, together with the occurrence of chromatin bridges and ring‐shaped quadrivalents, was interpreted as evidence of a high degree of homology among the four associated chromosomes, supporting an autopolyploid origin (Zeid et al., 2011). However, structural chromosome alterations observed in *Eragrostis* have also been considered indicative of segmental allopolyploidy (Vorster & Liebenberg, 1977). Together, these cytogenetic, reproductive, and genomic features make *E. curvula* an excellent model for studying genetic factors associated with polyploidization and shifts in reproductive mode.

Recent studies have highlighted the need to develop genomic and pangenomic resources to fully explore the genetic diversity of orphan crop species (Hu et al., 2025). In *E. curvula*, the assembly of the high‐quality reference genome of the sexual diploid cultivar Victoria was a major advance (Carballo et al., 2019). Building on this resource, an iterative assembly approach was later used to generate a pangenome that included cv. Victoria and the apomictic tetraploid cv. Tanganyika INTA (Carballo et al., 2023). However, a pangenome based on only two accessions cannot capture the species’ full intraspecific diversity.

Here, we assembled a panel of *E. curvula* accessions spanning ploidy levels from 2x to 7x and reproductive modes ranging from sexual to facultative or fully apomictic. Building on the diploid reference genome of cv. Victoria (Carballo et al., 2019) and the iterative mapping and assembly method, we generated a whole‐genome sequencing (WGS)‐based linear pangenome by incorporating genomic variation from 10 additional accessions. Although graph‐based and de novo approaches may provide higher resolution (Du et al., 2025; Liu et al., 2025), the availability of a high‐quality reference genome made the linear approach a suitable and cost‐effective strategy for expanding the reference gene pool across diverse cytotypes. The resulting pangenome enabled the expansion of the previously available genomic information as well as the identification of genes non‐annotated before. The identification of gene PAVs and genome‐wide single‐nucleotide polymorphisms (SNPs) among the selected cytotypes. Moreover, PAV analysis enabled the identification of two distinct candidate gene groups, respectively associated with polyploidy and apomixis, while genome‐wide SNP analysis provided insights into the evolutionary relationships among cytotypes. Together, these findings provide an important basis for future functional validation of the identified candidate genes associated with polyploidy and apomixis and represent the first genome‐wide assessment of genetic diversity in *E. curvula* based on genomic sequence data.

**Core Ideas**

- We built the first *Eragrostis curvula* pangenome across a panel of cytotypes, expanding the species’ reference gene pool.
- Cytotypes with different ploidy levels and reproductive modes exhibit extensive gene content variation.
- Gene groups associated with polyploidy and apomixis were identified through gene presence/absence variation analysis.
- Single‐nucleotide polymorphism (SNP) markers enabled inference of phylogenetic relationships among cytotypes.

## 2 MATERIALS AND METHODS

### 2.1 Plant material

The pangenome was built from the high‐quality genome assembly of the diploid (2x) sexual cv. Victoria (Carballo et al., 2019; NCBI BioProject PRJNA508722), together with WGS Illumina short‐read data from 10 *E. curvula* accessions (PI208214, PI299920, OTA‐S, Bahiense, Ermelo, Don Luis, Tanganyika USDA, TUNS9355, Don Eduardo, and Don Pablo) (Table S1). These accessions were selected from the germplasm available at CERZOS‐CONICET (https://cerzos.conicet.gov.ar/) to form a panel of *E. curvula* cytotypes that included distinct ploidy levels and reproductive modes, thus capturing a substantial part of the intraspecific diversity of the species. Plants were grown in 10‐L pots under greenhouse conditions at CERZOS‐CONICET following the natural spring photoperiod (approximately 16 h) in Bahía Blanca, Argentina (38°42′ S, 62°16′ W). The previously sequenced cytotype Tanganyika INTA (Carballo et al., 2023; NCBI BioProject PRJNA914706) was not included in the new pangenome assembly, as its higher sequencing depth (>100x) and longer read length (2 × 250 bp) would have introduced a bias in the iterative pangenome assembly (Table S1). However, it was retained for downstream comparative analyses, expanding the overall cytotype panel considered in this study to 12 accessions (Table S1).

### 2.2 Flow cytometry

For ploidy assessment, mature leaves were collected from *E. curvula* plants. About 150 mg of fresh tissue was processed to prepare nuclear suspensions using the CyStain PI Absolute P kit (Sysmex), with minor modifications to a previously described method (Doležel et al., 2007). Specifically, nuclear suspensions were incubated with 1.8 µL RNase and 3.6 µL propidium iodide (Sysmex) at 4°C for 30 min before analysis with a FACSCalibur flow cytometer (BD Biosciences). The relative DNA content was estimated from the fluorescence intensity of G1 peaks, and ploidy levels were inferred by comparing the G1 peak position of each sample with those of diploid and/or tetraploid internal standards (Victoria and OTA‐S, respectively). For diploid genotypes, OTA‐S was used as the internal standard; for tetraploids, Victoria; and for polyploids, both OTA‐S and Victoria were used.

### 2.3 DNA extraction and sequencing

Genomic DNA was isolated from 80 mg of fresh leaf tissue using the cetyltrimethylammonium bromide method, as previously described for *E. curvula* (Carballo et al., 2023; Meier et al., 2011). WGS libraries were prepared from the extracted DNA following Illumina protocols. DNA sequencing was performed on the NovaSeq PE150 platform with 2 × 150 bp paired‐end reads. Estimated whole‐genome coverage for each accession was calculated by dividing the total number of sequenced bases by the expected genome size of the corresponding cytotype, thereby accounting for differences in ploidy level. Per‐accession coverage values are reported in Table S1. The data generated for this project are available in the Sequence Read Archive under BioProject ID PRJNA1321158.

### 2.4 Pangenome assembly

The pangenome was assembled using an iterative approach (Carballo et al., 2023; Golicz, Bayer, et al., 2016; Montenegro et al., 2017; Ruperao et al., 2021; Zhao et al., 2020), based on a mapping‐and‐assembly methodology. At each iteration, raw Illumina paired‐end reads (https://www.ncbi.nlm.nih.gov/sra/?term=PRJNA1321158) were aligned to the current reference using Bowtie2 version 2.3.4 (http://bowtie‐bio.sourceforge.net) with the following parameters: –local ‐p 30 ‐I 0 ‐X 1000. Alignments were converted to BAM (Binary Alignment Map) format and sorted using SAMtools version 1.15.1 (https://github.com/samtools/samtools). Unmapped reads were extracted from the resulting BAM files using SAMtools flag‐based filtering (‐f 4) and used for de novo assembly with MaSuRCA version 3.2.8, setting the cgwErrorRate parameter to 0.15 (https://github.com/alekseyzimin/masurca). Mapped reads used for insert‐size estimation were filtered using a mapping quality threshold of Mapping Quality (MAPQ) ≥ 10, and insert‐size metrics were calculated with Picard Tools (https://broadinstitute.github.io/picard/). The resulting de novo assemblies were iteratively incorporated into the growing pangenome. To ensure sequence accuracy, assembled sequences were screened for plastid contamination and redundancy, following the filtering strategy previously described for *E. curvula* and other species using the same pangenome assembly strategy (Carballo et al., 2023; Hurgobin et al., 2018; Ruperao et al., 2021). Thus, the assembled sequences were aligned against the NCBI Viridiplantae plastid database to discard potential contamination using the BLAST alignment tool (Altschul et al., 1990). Then, to remove redundancy, the assembly was self‐compared using NUCmer from the MUMmer 4.0.0 beta 2 package (Marçais et al., 2018). Sequences showing ≥90% identity and ≥90% alignment coverage were considered redundant and removed. These thresholds were adopted from previous iterative pangenome studies using comparable filtering strategies (Carballo et al., 2023; Ruperao et al., 2021). The final pangenome was constructed over 10 iterations, starting from diploid (2x) sexual cytotypes and progressing to heptaploid (7x) apomictic cytotypes (Table S1). The pangenome assembly and annotation are available in the Zenodo repository under https://doi.org/10.5281/zenodo.18554196.

### 2.5 Pangenome annotation

Gene annotation was carried out using MAKER software version 3.01.02 (http://www.yandell‐lab.org/software/maker.html). Transcript evidence, including RNA‐Seq data from *Eragrostis tef* (NCBI BioProject PRJNA525065) and floral tissues of multiple *E. curvula* polyploid apomictic accessions (NCBI BioProjects PRJNA640521 and PRJNA358210), was used in the initial annotation iteration. These RNA‐Seq reads were aligned to the genome with STAR version 2.7.3a (parameters: –outSAMtype BAM SortedByCoordinate –readFilesCommand zcat –outFilterScoreMinOverLread 0.33 –outFilterMatchNminOverLread 0.33; https://github.com/alexdobin/STAR), and StringTie version 2.1.4 with default parameters (https://ccb.jhu.edu/software/stringtie/) was used to generate a Gene Transfer Format file to be used in MAKER. Ab initio predictions were integrated in MAKER using SNAP (2006‐07‐28) (https://github.com/KorfLab/SNAP) and AUGUSTUS version 3.3.3 (https://bioinf.uni‐greifswald.de/augustus/) with models trained through iterative rounds. The final genome annotation was obtained after three iterative runs of MAKER. Repetitive elements were masked with LTR Retriever version 2.9.6 using default parameters (Ou et al., 2018), and the completeness of the annotated pangenome was evaluated using Benchmarking Universal Single‐Copy Orthologs (BUSCO) version 3.0.1 with the liliopsida_odb10 database (Simão et al., 2015). Functional annotation was performed by sequence similarity searches against curated plant protein datasets. Predicted protein sequences were queried with BLASTp (BLAST+, version 2.11.0; https://ncbiinsights.ncbi.nlm.nih.gov/2020/11/12/blast‐2‐11‐0/) against (i) the NCBI nr plant protein database (available at https://www.ncbi.nlm.nih.gov/refseq/); (ii) the *Oryza sativa* STRING v12 protein dataset; and (iii) the *A. thaliana* STRING 12 protein dataset (available at https://string‐db.org). The best hit was used as the reference annotation based on sequence identity and alignment coverage.

### 2.6 Gene presence/absence detection

Paired‐end Illumina reads (150 bp for the 10 accessions and 250 bp for Tanganyika INTA) were aligned to the final pangenome assembly using Bowtie2 version 2.3.4 (http://bowtie‐bio.sourceforge.net). PAV was inferred using SGSGeneLoss version 0.1 with the default parameters (Golicz et al., 2015). Genes were classified as core when present in all accessions and as variable or accessory when absent in at least one accession. Variable genes were then classified according to their distribution across ploidy levels and reproductive modes. For the ploidy analysis, genes were classified as present or absent in each ploidy group (2x, 4x, 6x, and 7x) based on whether they were detected in all or none of the accessions in that group. The polyploid‐associated gene set was defined as genes absent from all diploid accessions and present in all tetraploid, hexaploid, and heptaploid accessions: *G*_poly_ = (*Ab*_2x_ ∩ *Pr*_4x_ ∩ *Pr*_6x_ ∩ *Pr*_7x_), where *Ab* indicates genes absent from all accessions of a given ploidy group and *Pr* indicates genes present in all accessions of that group. For reproductive‐mode comparisons, genes were classified as present when detected in at least one accession in a group and lost when absent from at least one accession of that group. Apomixis‐associated genes were defined as *G*_apo_ = (*Pr*_apo_ ∩ *Ab*_sex_)—(*Ab*_apo_ ∪ *Pr*_sex_), whereas sexual‐associated genes were defined as *G*_sex_ = (*Pr*_sex_ ∩ *Ab*_apo_)—(*Ab*_sex_ ∪ *Pr*_apo_). Gene set intersections were visualized in R using the ggvenn package (v0.1.19). Differences in the number of present genes among diploid (2x), tetraploid (4x), and hexaploid (6x) accessions were evaluated using Welch's *t*‐tests (Ruxton, 2006). The heptaploid (7x) group was excluded from these comparisons because it was represented by a single accession. Differences between diploid (2x) and polyploid (4x, 6x, and 7x) accessions were also evaluated using Welch's *t*‐test. The relationship between ploidy level and gene number was assessed using linear regression (Draper & Smith, 1998), including diploid, tetraploid, and hexaploid accessions. Mean ± SD (standard deviation) values were calculated for each ploidy group. Statistical analyses were performed in Python v3.12 using SciPy v1.13.1 (Virtanen et al., 2020), with *p* < 0.05 considered statistically significant.

### 2.7 Presence/absence validation

Qualitative reverse transcription polymerase chain reaction (RT‐PCR) analysis was used to validate PAV predictions. Total RNA was extracted from spikelets following the manufacturer's instructions using the SV Total RNA Isolation System (Promega). First‐strand cDNA (complementary DNA) synthesis was performed using M‐MuLV Reverse Transcriptase (New England Biolabs) from independent RNA samples corresponding to each accession. Gene‐specific primers were designed exclusively within exonic regions using Primer‐BLAST (Ye et al., 2012) (Table S2). Degenerate nucleotides (W, R, and Y) were added to primer sequences when needed. PCR reactions were carried out using cDNA from all accessions included in the pangenome analysis. The *UBICE* gene was used as an internal control for RT‐PCR amplification (Carballo et al., 2023), using a previously developed primer pair (Garbus et al., 2017). In addition, four primer pairs previously developed for the screening of apomixis‐associated (*EcAPO*) candidate genes in *E. curvula* (Garbus et al., 2026) were used for an in silico PCR analysis (Kalendar et al., 2024). Primer sequences are provided in Table S2. First, primers were aligned against the pangenome sequences using BLASTn with the ‐task blastn‐short option (BLAST+, version 2.11.0; https://ncbiinsights.ncbi.nlm.nih.gov/2020/11/12/blast‐2‐11‐0/). Then, a custom Perl script was used to predict the corresponding amplicons. Finally, amplicon coordinates were intersected with the pangenome annotation to identify any annotated gene models overlapping the predicted amplicons.

### 2.8 Gene Ontology and transcription factor analyses

Genes identified by PAV analysis as specifically associated with cytotypes with ploidy levels greater than 2x or with apomictic reproduction were subjected to Gene Ontology (GO) enrichment analysis. Protein sequences were functionally annotated with EggNOG‐mapper (Cantalapiedra et al., 2021; version 2.1.13) using the Viridiplantae taxonomic scope for orthology assignment and functional annotation. GO enrichment analysis was performed in R using the enricher function implemented in the clusterProfiler package (G. Yu et al., 2012; version 3.23.1), while GO term names were associated with EggNOG GO IDs using the GO.db annotation package (https://bioconductor.org/packages/GO.db, version 4.6). The enricher function was run with pvalueCutoff = 0.05, qvalueCutoff = 0.05, and pAdjustMethod = “BH,” using the complete pangenome gene set, comprising 97,082 gene models, as the reference background. For visualization, the top 20 enriched GO terms were shown in a global GO enrichment bar plot. Transcription factors (TFs) and transcriptional regulators (TRs) within the same PAV‐defined gene sets were identified and classified into families using iTAK (Zheng et al., 2016; version 1.6).

### 2.9 Short‐variant calling

SNPs and insertions/deletions (InDels) were identified using the sequencing data employed for the PAV analysis. Reads were aligned to the pangenome reference using the BWA‐MEM algorithm implemented in BWA v0.7.17‐r1188 (https://github.com/lh3/bwa), with the ‐M and ‐R parameters, and variants were detected following the Genome Analysis Toolkit (GATK) Best Practices workflow (McKenna et al., 2010; v4.2.1.0). Briefly, short variants were first called independently for each accession using GATK HaplotypeCaller in GVCF (Genomic Variant Call Format) mode, generating one genomic VCF (gVCF) file per accession. The individual gVCFs were subsequently combined using GATK CombineGVCFs and jointly genotyped with GATK GenotypeGVCFs to produce a single multi‐sample Variant Call Format (VCF) containing all accessions. BCFtools v1.16 (https://github.com/samtools/bcftools) was used to retain biallelic SNPs and remove InDels, whereas VCFtools v0.1.16 (https://vcftools.github.io/) was employed to evaluate the fraction of missing genotypes and sequencing depth for each accession. For phylogenetic analysis, biallelic SNPs (‐m2 ‐M2 –types snps) with no more than three missing genotypes (‐e ‘N_MISSING > 3’) were retained using BCFtools. To avoid bias from linked variants and selection, the VCF file was also first annotated with SnpEff (Cingolani et al., 2012), and the bcftools view ‐i option was used to retain neutral and intergenic sites. Then, to retain only independent sites, linked SNPs were pruned with PLINK software (Chang et al., 2015; v.1.9.0‐b7.7) using the following parameters: sliding window = 50, step size = 10, and *r* ^2^ = 0.2. Finally, the resulting VCF file was converted to PHYLIP format with the vcf2phylip script (Ortiz, 2019), and a phylogenetic network was produced with SplitsTree (Huson & Bryant, 2024) using p‐distance and the Neighbor‐Net algorithm. SNP‐call heterozygosity was estimated for each accession using scikit‐allel v1.3.8 (https://scikit‐allel.readthedocs.io/) as the proportion of heterozygous genotype calls among all non‐missing SNP genotype calls. Genome‐wide allele frequency (AF) distributions were examined following the “Determining ploidy 1” workflow described in the vcfR documentation (Knaus & Grünwald, 2017). In addition, the mean sequencing depth across the heterozygous SNPs retained for each accession was calculated.

## 3 RESULTS

### 3.1 Ploidy variation in the *E. curvula* panel

**FIGURE 1:**
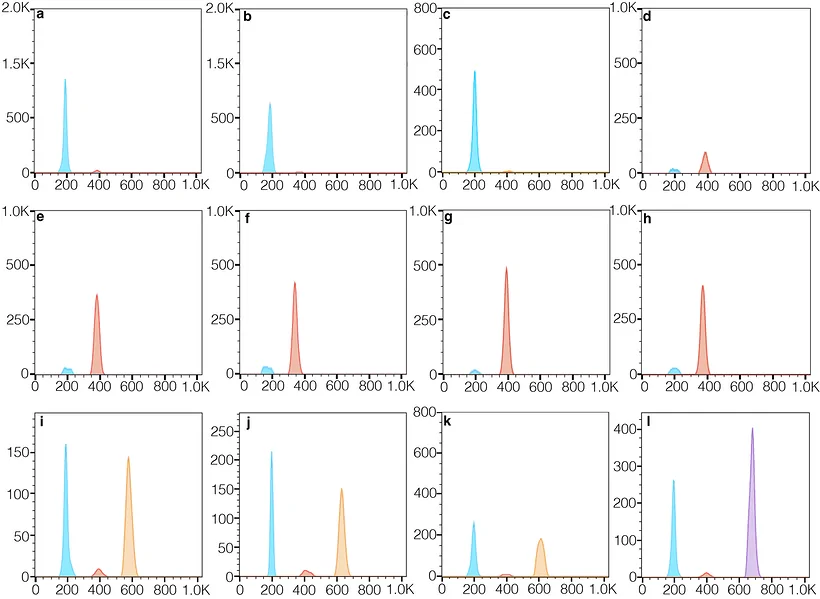
Flow cytometry histograms. The *x*‐axis represents fluorescence intensity (relative DNA content), and the *y*‐axis indicates the number of nuclei. Each panel corresponds to one accession: (a) PI299920, (b) PI208214, (c) Victoria, (d) Tanganyika INTA, (e) Tanganyika USDA, (f) Bahiense, (g) OTA‐S, (h) Ermelo, (i) Don Luis, (j) TUNS9355, (k) Don Eduardo, and (l) Don Pablo. Colors indicate ploidy levels: 2x (light blue), 4x (red), 6x (orange), and 7x (purple). Victoria (2x) and OTA‐S (4x) were used as internal reference standards.

From different accessions of *E. curvula* available in the CERZOS‐CONICET germplasm collection, a specific panel of 12 cytotypes was selected based on available information (Carballo, Zappacosta, Selva, et al., 2021; Carballo et al., 2023; Meier et al., 2011), including ploidy level and reproductive mode. The ploidy level of plants for each selected genotype was verified by flow cytometry (Figure 1). Peaks at the diploid level (2x relative DNA content) were observed for the sexual PI208214, PI299920, and cv. Victoria genotypes, confirming their diploid status. Peaks at 4x were detected for the fully apomictic Tanganyika USDA, the facultative apomictic Tanganyika INTA, Bahiense, and Ermelo, as well as for the sexual OTA‐S, confirming their tetraploid status. Peaks at 6x were observed for the facultative apomictic Don Luis, TUNS9355, and Don Eduardo, confirming that they are hexaploid. A major peak corresponding to 7x relative DNA content was detected only for the apomictic accession Don Pablo, which was therefore classified as heptaploid. In the hexaploid and heptaploid flow cytometry plots, two additional peaks corresponding to the diploid and tetraploid controls were observed (Figure 1). Therefore, the panel included three diploids, five tetraploids, three hexaploids, and one heptaploid accession. These results confirm that, despite its limited size, the assembled panel captures distinct ploidy levels and reflects much of the intraspecific ploidy diversity present in *E. curvula*.

### 3.2 Pangenome construction

**FIGURE 2:**
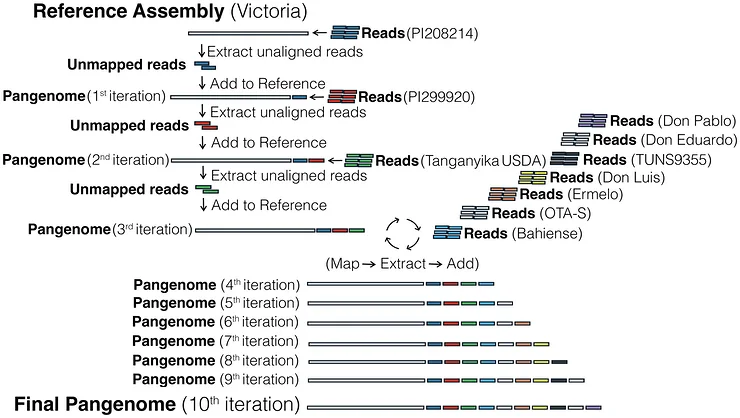
Iterative mapping and assembly workflow used to construct the *Eragrostis curvula* pangenome. Illumina paired‐end reads from each accession were sequentially mapped to the cv. Victoria reference genome. Unmapped reads were extracted and assembled to generate an updated pangenome, which served as the reference for subsequent iterations. Accessions were incorporated in the order shown, from diploid to heptaploid cytotypes, each contributing novel sequences. The progressive increase in pangenome size across iterations is illustrated.

The availability of a high‐quality reference genome (Carballo et al., 2019) enabled the construction of a WGS‐based pangenome through iterative mapping and assembly of short Illumina reads generated for each accession (on average, ∼25.8 Gb of 2 × 150 bp paired‐end reads; Table S1). As a result, the pangenome was linear and combined the Victoria genome assembly with new genomic sequences from different cytotypes of the species (Figure 2).

On average, each iteration of the process added 29.6 Mb, for a total of approximately 296 Mb across 10 iterations (Figure S1; Table S3). Thus, before the final filtering step, the iterative assembly increased the genome size from 603 Mb for the Victoria reference to approximately 899 Mb for the newly assembled pangenome. The largest increases in cumulative pangenome size occurred when unmapped sequences from the first tetraploid accession (Tanganyika USDA) and the first hexaploid accession (Don Luis) were incorporated, adding approximately 76 and 122 Mb, respectively. Subsequent accessions of the same ploidy levels generally contributed less new sequence. Among the remaining tetraploids, Bahiense, OTA‐S, and Ermelo added approximately 16.2, 10.6, and 0.05 Mb, respectively. Similarly, after the increase in the size of the pangenome associated with Don Luis, TUNS9355 added only 0.003 Mb, followed by a further increase of 37.1 Mb with Don Eduardo, whereas the heptaploid accession Don Pablo contributed only 0.002 Mb (Figure S2; Table S3). Considering the cumulative contribution of accessions within each ploidy level, diploid, tetraploid, and higher polyploid (>4x) groups added 33.7, 103.3, and 159.5 Mb, respectively. Within the tetraploid group, the three apomictic accessions collectively contributed 92.7 Mb, nearly nine times more sequence than the single sexual accession OTA‐S. When accessions were grouped by reproductive mode regardless of ploidy level, sexual accessions contributed a total of 44.3 Mb, whereas apomictic accessions contributed 252.1 Mb to pangenome growth (Table S3). The final filtering step removed 4054 sequences, including 4019 redundant and 35 putative plastid‐derived sequences, corresponding to a total of 4,280,094 bp. After filtering, the final pangenome spanned approximately 895 Mb with an N50 of 23.8 Mb (Table S4).

### 3.3 Gene annotation

A total of 97,082 gene models were predicted after three rounds of iterative gene model annotation with MAKER, nearly doubling the number reported for the Victoria reference (56,469 gene models; Carballo et al., 2019). Repetitive elements accounted for 24% of the assembly, a proportion lower than in the single‐reference genome (i.e., the Victoria high‐quality reference genome). This was expected because the iterative mapping and assembly approach increased the final assembly size from 603 Mb in Victoria to 895 Mb by adding more unique sequences into the scaffolds rather than additional repetitive regions, while the redundancy‐filtering criteria further reduced the incorporation of sequences already represented in the pangenome. BUSCO assessment using the liliopsida_odb10 dataset showed 95.7% completeness, including 66.5% single‐copy and 29.2% duplicated orthologs, with only 2.2% fragmented and 2.1% missing (Table S4). These results are consistent with an iterative multi‐cytotype pangenome, where the proportion of duplicated orthologs is attributable to the representation of polyploid genomes and accessory genomic sequences.

### 3.4 Gene presence/absence detection

PAV analysis revealed extensive variation in gene content among *E. curvula* cytotypes. In total, 69,438 genes were classified as core because they were shared by all accessions, whereas 27,644 were classified as variable because they were present in some accessions but absent in others (Figure S3). Gene content generally increased with ploidy, with higher ploidy accessions containing more genes than tetraploids, and tetraploids containing more genes than diploids. However, substantial variation was also observed within the tetraploid group. The apomictic tetraploids Ermelo and Bahiense and the sexual tetraploid OTA‐S contained fewer genes (82,774, 83,247, and 83,213, respectively), whereas the apomictic tetraploids Tanganyika USDA and Tanganyika INTA showed higher gene counts (88,414 and 90,546, respectively) (Table S5). These results indicate that gene content variation occurs not only among ploidy levels but also within the same ploidy class. Mean gene counts (mean ± SD) were 77,365 ± 1480.9 in diploid accessions, 85,638.8 ± 3591.5 in tetraploid accessions, and 93,923 ± 45.7 in hexaploid accessions, whereas the single heptaploid accession contained 93,981 genes (Table S5). Welch's *t*‐tests also showed significant differences between diploid and tetraploid, diploid and hexaploid, and tetraploid and hexaploid accessions (*p* < 0.01 in all comparisons). Polyploid accessions, including the heptaploid accession, contained significantly more genes than diploid accessions (Welch's *t*‐test, *p* < 0.0001). Linear regression further showed a significant positive relationship between ploidy level and gene number (*R* ^2^ = 0.88, *p* < 0.00001) (Table S5).

### 3.5 Identification of genes associated with polyploidy and apomixis

**FIGURE 3:**
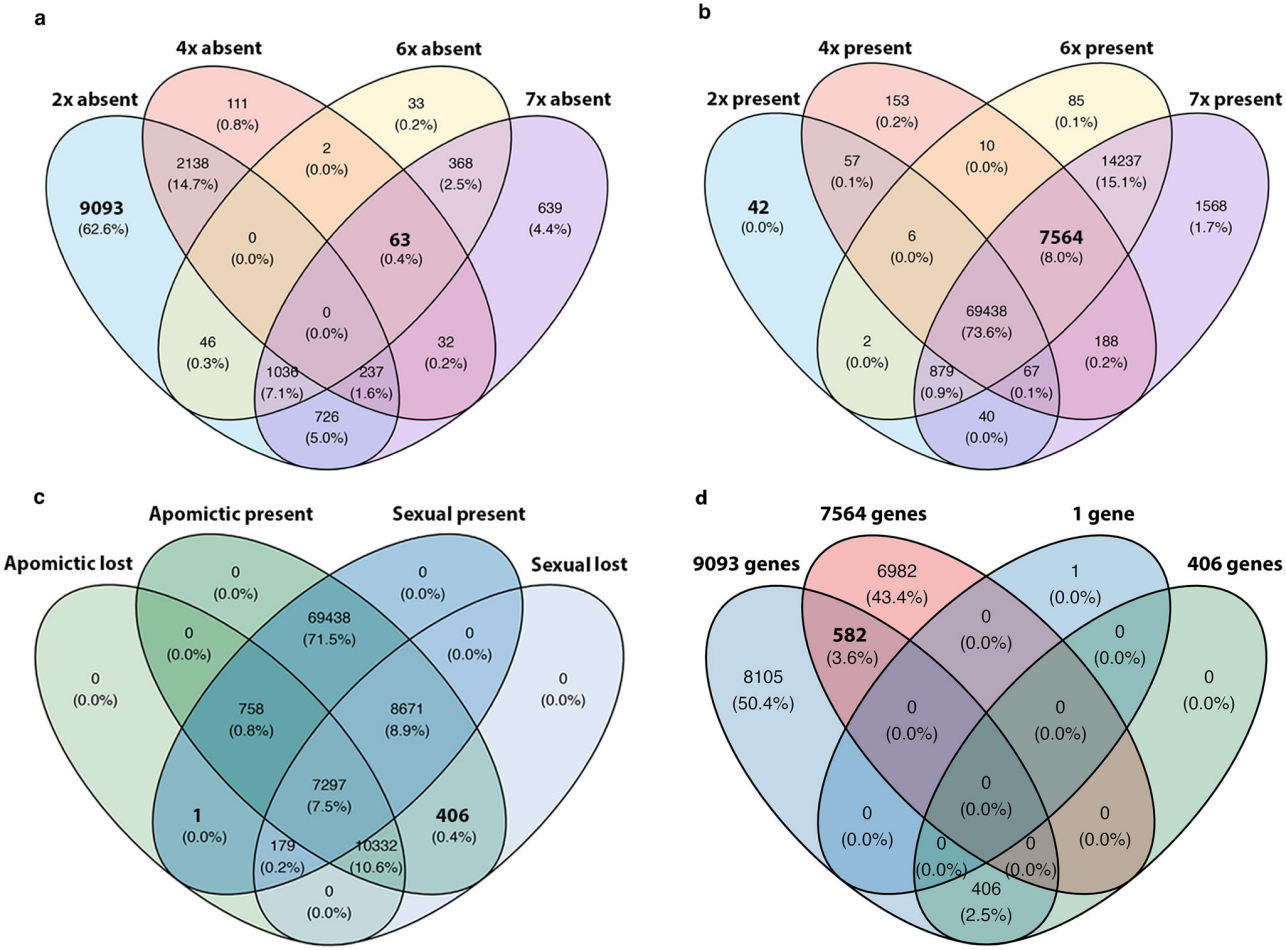
Gene presence/absence variation (PAV) across ploidy levels and reproductive modes. Venn diagrams showing gene sets classified according to their distribution across cytotypes. (a) Genes absent in all accessions within each ploidy group, including 9093 genes absent in all diploid accessions and present in at least one polyploid accession. (b) Genes present in all accessions within each ploidy group, including 7564 genes present in all polyploid accessions and absent in at least one diploid accession. (c) Exclusive intersections of reproductive‐mode gene sets, identifying 406 apomixis‐associated genes and one sexual‐associated gene. (d) Joint intersection of ploidy‐ and reproductive‐mode‐related gene sets, identifying 582 genes consistently absent in all diploid accessions and present in all polyploid accessions, with no overlap with the apomixis‐ or sexual‐associated gene sets.

PAVs were used to examine gene distribution across ploidy levels and reproductive modes (Figure 3). Using the presence/absence criteria described above, 63 genes were absent in all polyploid cytotypes and present in at least one diploid accession, whereas 9093 genes were absent in all diploids and present in at least one polyploid accession (Figure 3a). Conversely, 7564 genes were present in all polyploids and absent from at least one diploid accession, and 42 genes were present in all diploid accessions and absent from at least one polyploid accession (Figure 3b). For reproductive mode, four gene sets were considered: genes detected in at least one apomictic accession (apomictic present), genes absent from at least one apomictic accession (apomictic lost), genes detected in at least one sexual accession (sexual present), and genes absent from at least one sexual accession (sexual lost) (Figure 3c). The exclusive intersection of these four sets identified 406 genes associated with the apomictic phenotype that were present in all apomictic accessions and absent from all sexual accessions (Figure 3c; Table S6). The reciprocal exclusive intersection identified a single gene associated with the sexual phenotype, which was present in all sexual accessions and absent in all apomictic accessions (Figure 3c). Finally, genes absent in all diploid accessions and present in at least one polyploid accession (9093 genes), genes present in all polyploid accessions and absent in at least one diploid accession (7564 genes), genes associated with the apomictic phenotype (406 genes), and the unique gene associated with the sexual phenotype were included in the same intersection analysis (Figure 3d). The intersection of the two ploidy‐related sets identified 582 genes (9093 ∩ 7564 = 582; Table S6) that were consistently absent in all diploid accessions and present in all polyploid accessions and were therefore defined as polyploid‐associated genes. None of these 582 genes overlapped with either the 406 genes associated with the apomictic phenotype or the single gene associated with the sexual phenotype (Figure 3d).

### 3.6 In vitro and in silico validation of PAVs

A subset of genes identified by PAV analysis as uniquely present in apomictic cytotypes was subjected to in vitro validation by qualitative RT‐PCR (Figure S4). In many cases, gene validation was laborious and time‐consuming because working with numerous sexual and apomictic accessions that differed not only in reproductive mode but also in ploidy level increased genomic complexity. However, a clear amplification was obtained for one apomixis‐specific gene that showed similarity to the *A. thaliana* orthologue *PVA31* (45.08% amino acid identity), which encodes a vesicle‐associated protein putatively involved in intracellular vesicle trafficking. Qualitative RT‐PCR analysis showed amplification of the *PVA31*‐like transcript exclusively in apomictic individuals, whereas no amplification was detected in sexual accessions (Figure S4a); the housekeeping gene *UBICE* (UBIQUITIN CONJUGATING ENZYME TRANSCRIPT) was amplified in all samples (Figure S4b). Regarding the in silico PCR results, targets of the *EcAPO* primers were identified within PAV‐derived gene sets. *EcAPO1*‐specific primers matched a gene model annotated as a hypothetical protein and included among the 406 apomixis‐associated genes, whereas *EcAPO2*‐specific primers mapped to a *CWC22*‐like pre‐mRNA (precursor messenger RNA) splicing factor within the same gene set. *EcAPO3*‐ and *EcAPO4*‐specific primers targeted an A‐type cyclin and an F‐box protein, respectively, both included among the 582 polyploid‐associated genes. The predicted amplicons, the corresponding gene models, and their functional annotations are listed in Table S7.

### 3.7 GO enrichment analysis of polyploidy‐ and apomixis‐associated genes

**FIGURE 4:**
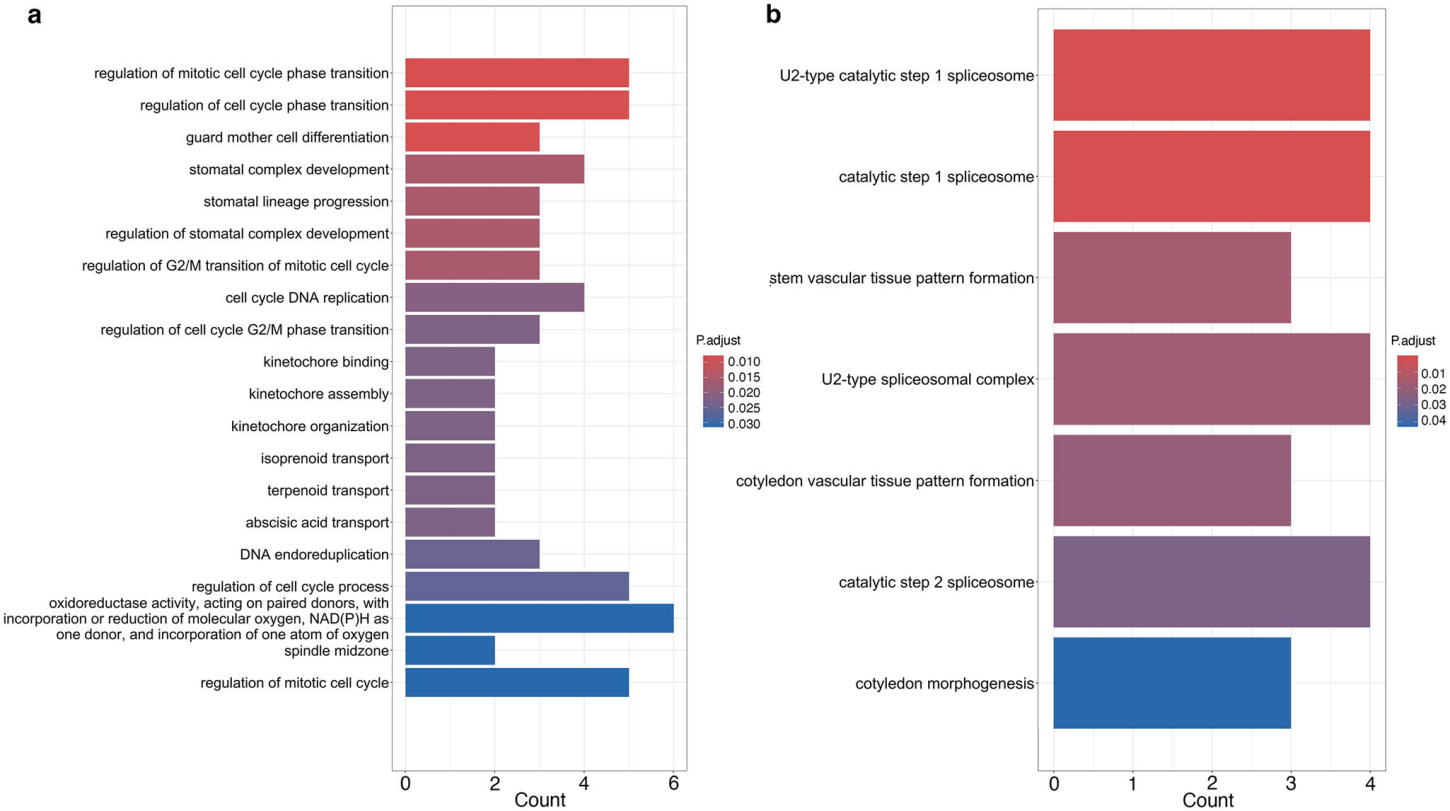
Functional classification of genes associated with ploidy level and reproductive mode. Gene Ontology (GO) enrichment analysis of candidate gene sets. (a) Enriched GO terms for the 582 ploidy‐associated genes. (b) Enriched GO terms for apomixis‐associated genes. The top 20 significantly enriched GO terms are shown for each analysis. Bars represent the number of genes assigned to each GO term, and colors indicate adjusted *p*‐values.

Functional differences between genes associated with polyploidy and those associated with apomixis were investigated using GO enrichment analysis. Among polyploidy‐associated genes, the most significantly enriched GO terms were related to cell‐cycle regulation and processes associated with genome stability, including mitotic phase transitions (G2/M), kinetochore‐ and spindle‐associated functions, and DNA replication (Figure 4a; Table S8). These categories were linked to genes annotated as A‐type cyclins (e.g., *CycA2*), kinesin motor proteins (e.g., *KIN‐7*), and replication protein A subunits (e.g., *RPA2*; also known as *RPA32* based on its molecular weight). Among these genes, the Cyclin‐A2‐like gene model targeted by the *EcAPO3* primers contributed to several enriched GO categories related to cell‐cycle regulation, DNA replication, endoreduplication, and stomatal development (Tables S7 and S8). The *EcAPO4* target was also included in the polyploidy‐associated gene set but did not contribute to any significantly enriched GO category. A‐type cyclins regulate cyclin‐dependent kinase (CDK) activity and play a central role in cell‐cycle control (Renaudin et al., 1996). Kinesin motor proteins participate in chromosome segregation and mitotic progression (Kriechbaum & Müller, 2026), while replication protein A subunits, including *RPA2*, are involved in DNA replication and response to DNA damage (Chowdhury et al., 2021). *CycA2*‐like genes were also associated with other enriched GO terms related to developmental processes, including stomatal complex development, guard mother cell differentiation, stomatal lineage progression, and regulation of stomatal complex development (Figure 4a; Table S8). In this case, the enriched categories were consistent with the role of A‐type cyclins in coordinating cell‐division programs during stomatal development (Pillitteri et al., 2011). Additional enriched terms included isoprenoid transport, terpenoid transport, and abscisic acid transport (Figure 4a; Table S8) and were linked to genes annotated as MATE efflux transporters (e.g., DETOXIFICATION 51) and members of the ABCG transporter family (ABCGs). MATE efflux transporters are involved in the regulation of plant development by controlling phytohormone transport and tip‐growth processes (Upadhyay et al., 2019), while ABCG transporters mediate the transport of secondary metabolites and phytohormones and contribute to plant growth and development (Do et al., 2018).

Apomixis‐associated genes showed a marked predominance of GO categories related to RNA processing and post‐transcriptional regulation, with significantly enriched terms for the catalytic Step 1 spliceosome, catalytic Step 2 spliceosome, and the U2‐type spliceosomal complex (Figure 4b; Table S9). These categories were linked to genes annotated as *CWC22*‐like pre‐mRNA splicing factors. *CWC22* is an essential splicing factor required for exon junction complex assembly and efficient mRNA splicing (Steckelberg et al., 2012). One of the *CWC22*‐like gene models identified among the apomixis‐associated genes corresponded to the target of the *EcAPO2* primers and contributed to the enriched spliceosome‐related GO categories (Tables S7 and S9). By contrast, the *EcAPO1* target was included in the apomixis‐associated PAV‐derived gene set but did not contribute to any significantly enriched GO category. Additional enriched GO terms were related to developmental processes, including stem vascular tissue pattern formation, cotyledon vascular tissue pattern formation, and cotyledon morphogenesis (Figure 4b; Table S9). These categories included genes annotated as ABC transporter G family members (ABCG) (Figure 4b; Table S9). Together, these results suggest that RNA‐processing and spliceosome‐associated factors represent candidate components potentially involved in regulating reproductive development in *E. curvula* apomictic cytotypes.

More specifically, three A‐type cyclin‐like genes and four *CWC22*‐like genes were associated with the enrichment of cell‐cycle‐ and spliceosome‐related GO terms, respectively (Tables S8–S10). By inspecting the entire annotated pangenome, additional gene models that were either functionally related to these genes or that shared the same annotation were identified. Among these, a gene model homologous to *CDKG1*, a member of the CDK family whose members regulate cellular processes ranging from cell division to cell death (Malumbres, 2014), was identified among the polyploid‐associated genes (Tables S6 and S10), although it was not directly associated with any of the significantly enriched GO terms. In addition, a *CWC22*‐like gene not included in the set of 406 apomixis‐associated genes was detected only in Tanganyika USDA and Don Luis and was absent from all other accessions (Table S10).

### 3.8 Identification of TF/regulator families

The analysis of TFs and TRs identified TF/TR families shared between genes associated with polyploidy and apomixis, as well as families uniquely associated with each category (Figure S5; Tables S11 and S12). Among the shared TF families, FAR‐RED IMPAIRED RESPONSE 1 (FAR1) was the most represented, with seven genes in the polyploid category and three in the apomictic category. FAR1‐related proteins are transposase‐derived TFs that are crucial components of phytochrome A‐mediated far‐red light signal transduction and play important roles in plant growth and development (Ma & Li, 2018). Another TF family identified in both categories was the B3 family (one and two genes identified in the polyploid and apomictic categories, respectively). The B3 family is one of the most extensive plant‐specific TF families (Swaminathan et al., 2008) and includes subfamily members involved in embryogenesis, such as LEC2 (Stone et al., 2008) and FUS3 (Wu et al., 2020). In addition to TFs, TRAF (tumor necrosis factor receptor‐associated factors)‐type TRs were identified among genes associated with both polyploidy and apomixis (two and three TRAF genes in the polyploid and apomictic categories, respectively). TRAF proteins function as molecular adaptors or E3 ubiquitin ligases involved in signal transduction (Park, 2018; Xie, 2013) and have been implicated in plant immunity (S. Huang et al., 2016) and in megaspore mother cell specification and gametophyte development (Singh et al., 2017).

Among TF families identified by analyzing polyploid‐associated genes, the families included C2C2‐Dof (one gene), C2H2 (one gene), lateral organ boundaries (LOB)/ Lateral Organ domainLBD (one gene), Plant A/T‐rich sequence‐ and zinc (PLATZ) (one gene), and zinc finger‐homeodomain (ZF‐HD) (one gene) (Figure S5; Table S11). The DNA‐binding with one finger (DOF) TF family is plant‐specific and contains a conserved DOF domain with a C2C2 zinc finger that binds the 5′‐AAAG‐3′ cis‐element (Yanagisawa, 2004). It is involved in flowering time, metabolism, germination, vascular development, and stress responses (Fornara et al., 2009; Yanagisawa, 2004). C2H2‐type zinc finger proteins are involved in trichome initiation and abiotic stress responses (G. Han et al., 2020), while LOB/LBD TFs regulate lateral organ development and metabolic processes (Xu et al., 2016). PLATZ‐binding TFs are involved in plant growth, development, and adaptation to environmental changes (H. Zhang, Liu, et al., 2025), and ZF‐HD proteins regulate plant growth, development, and stress responses (J. Li et al., 2024).

TF families uniquely associated with apomixis‐related genes included AP2/ERF (two genes), myeloblastosis (MYB)/MYB‐related (two genes), and NAC (one gene) (Figure S5; Table S12). The AP2/ERF family is involved in development and stress responses (Gu et al., 2017) and includes BBM/PLT4, a key regulator of plant embryogenesis (Chen et al., 2022). The MYB TFs regulate metabolism, cell division and differentiation, plant development, hormone responses, and adaptation to biotic and abiotic stresses (D. Zhang, Zhou, et al., 2025). The NAC TFs (K. Han et al., 2023) play important roles in embryogenesis (Kunieda et al., 2008) and in stress responses (Takasaki et al., 2010) (Figure S5; Table S12).

### 3.9 Genome‐wide SNP analysis

**FIGURE 5:**
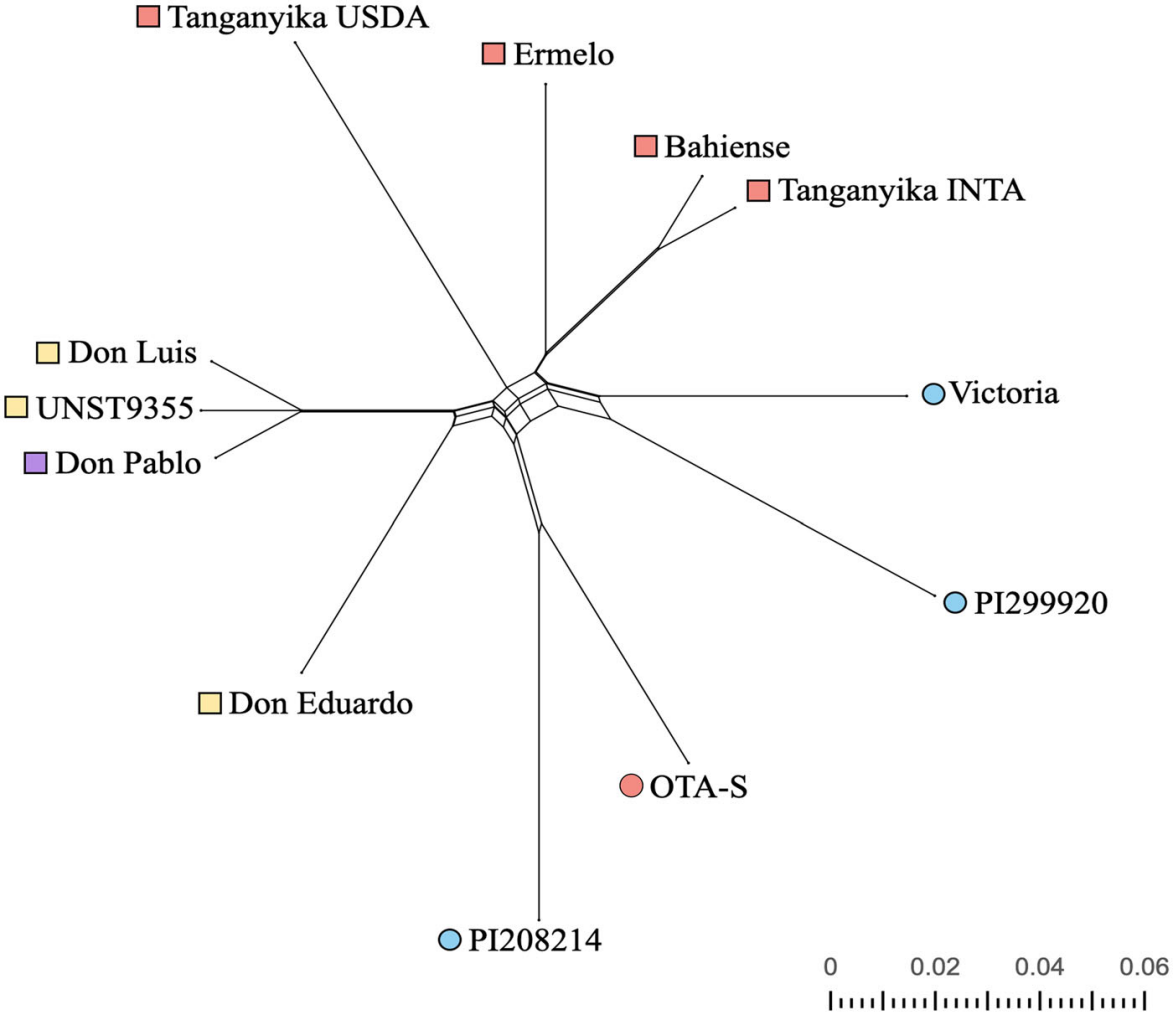
Genetic relationships among *Eragrostis curvula* accessions based on genome‐wide SNPs. Neighbor‐Net phylogenetic network inferred from 1,696,724 SNPs. Colors indicate ploidy levels: 2x (light blue), 4x (red), 6x (yellow), and 7x (purple). Shapes indicate reproductive mode: circles = sexual accessions, squares = apomictic accessions. The scale bar indicates genetic distance.

While PAV analysis, supported by in vitro and in silico tests, identified differences in gene content among the *E. curvula* accessions, genome‐wide SNP analysis enabled the determination of their genetic relationships. Short‐variant calling based on the same WGS data identified approximately 11 million InDels and more than 52 million SNPs before filtering (Table S13). After removing InDels and multiallelic sites, about 47 million biallelic SNPs were retained. Subsequently, only neutral, intergenic, unlinked SNPs with no more than three missing genotypes were retained, resulting in a final dataset of 1,696,724 sites (Tables S13 and S14). The Neighbor‐Net phylogenetic network generated from this dataset revealed the genetic relationships among the accessions (Figure 5). The high‐ploidy facultative apomictic accessions Don Luis, TUNS9355, and Don Pablo formed a closely related group, whereas Don Eduardo occupied a separate branch connected to the same region of the network. Tanganyika INTA and Bahiense formed a closely related pair, with Ermelo connected to the same branch. The sexual accessions OTA‐S (4x) and PI208214 (2x) were positioned relatively close to each other, sharing the same network, whereas Victoria (2x) and PI299920 (2x) formed a separate pair. Tanganyika USDA (4x, fully apomictic) formed a distinct branch within the network (Figure 5). Principal component analysis (PCA) supported these relationships, with PC1 and PC2 explaining 23.6% and 20.6% of the total variation, respectively (Figure S6). High‐ploidy accessions clustered closely, while Tanganyika INTA, Bahiense, and Ermelo occupied a nearby region of the PCA space. Victoria and PI299920 were clearly separated from the remaining accessions, whereas OTA‐S and PI208214 occupied more peripheral positions in the PCA space (Figure S6).

Genome‐wide SNP heterozygosity analysis showed that most accessions were predominantly homozygous at reference loci, whereas the proportions of heterozygous loci and homozygous alternative loci varied markedly among accessions (Figure S7; Table S15). High‐polyploid facultative apomictic accessions showed the highest SNP‐call heterozygosity (31.69%–33.49%) and the lowest homozygous reference fractions (60.65%–62.48%). To a lesser extent, the fully apomictic tetraploid Tanganyika USDA also showed high SNP‐call heterozygosity (27.82%) and a slightly higher homozygous reference fraction (65.69%) than the high‐polyploid accessions. In contrast, diploid sexual accessions showed the highest homozygous reference fractions (80.30%–89.16%) and the lowest SNP‐call heterozygosity (6.57%–9.61%), whereas the remaining tetraploid accessions showed SNP‐call heterozygosity ranging from 16.03% to 20.33% and homozygous reference fractions ranging from 74.94% to 79.61%.

When AF distributions at heterozygous SNP loci were examined across cytotypes, distinct AF profiles were observed among accessions (Figure S8). Average read depth across the heterozygous SNP sites used for AF analysis ranged from 17.9x in PI208214 to 144.8x in Tanganyika INTA, with most accessions showing values between approximately 25x and 42x (Table S16). Diploid accessions PI208214 and PI299920 showed AF distributions centered around 0.5. Tetraploid accessions Bahiense, Tanganyika INTA, OTA‐S, and Ermelo showed AF peaks at approximately 0.25, 0.5, and 0.75, although their profiles differed in resolution and relative peak heights. Tanganyika USDA showed a dominant AF peak centered around 0.5. High‐polyploid accessions exhibited more heterogeneous and multi‐peaked AF profiles, including peaks around 1/6, 1/4, 1/3, 1/2, 2/3, 3/4, and 5/6 (Figure S8).

## 4 DISCUSSION

Polyploidization is widely recognized as a key driver of diversification in grasses (Poaceae), and *E. curvula* is no exception. This species forms a classic agamic complex (Harlan & De Wet, 1963) comprising populations with different ploidy levels, in which increases in ploidy are often associated with a shift from sexual to apomictic reproduction. Although a chromosome‐scale genome assembly was a key genomic resource, the absence of the apomictic trait and, likely, the components controlling this region, along with the diploid nature, limited its utility for investigating polyploidy and apomixis. Here, we sequenced and assembled a pangenome from accessions with different ploidy levels and reproductive modes using a linear pangenome approach. This approach was chosen based on the success achieved in previous studies in *E. curvula* (Carballo et al., 2023) and because it is a cost‐effective method for identifying gene PAVs as well as short variants to study both polyploidy and apomixis.

In general, pangenomes such as the one assembled here can be classified as open or closed, depending on whether additional genomes continue to contribute novel sequences (Matthews et al., 2024). The previously available genomic data, derived from a diploid genome assembly and a two‐genotype‐based pangenome, were significantly increased in terms of genes and sequences. The contribution to sequence growth generally decreased as more individuals at the same ploidy level were included, consistent with diminishing returns (Figure S2; Table S3). Nonetheless, the hexaploid cytotype (Don Eduardo) that was added as the last during the mapping and assembly iterations still added 37.1 Mb, indicating that the increase in pangenome size was influenced not only by the ploidy level but also by the relative genetic divergence of each accession and the order of incorporation during the iterative mapping and assembly process. This may also explain the very limited contribution of the heptaploid Don Pablo (Table S3), which was incorporated as the final accession after most of its genomic sequences had already been represented in the growing pangenome through the previous assembly iterations. This behavior is probably related to its close relationship with the other accessions, as shown in the phylogenetic network (Figure 5).

PAV analysis revealed substantial variation in gene content among the selected *E. curvula* cytotypes, and qualitative RT‐PCR validation of the apomixis‐candidate *PVA31*‐like gene provided targeted experimental support for this PAV‐derived prediction. We selected a gene present in apomictic but absent in sexual accessions, since there is a single region associated with apomictic genotypes that controls the trait and may include genes with higher conservation levels (Gallardo et al., 2025; Zappacosta et al., 2019). Validation was initially limited to a small subset of candidate genes because PCR‐based validation of PAVs across multiple cytotypes proved technically complex. During primer design, candidate genomic regions frequently contained numerous accession‐specific SNPs at potential primer annealing sites, and the polyploid nature of *E. curvula* added complexity due to highly similar homologous and homeologous genomic regions. Indeed, in polyploids, particularly allopolyploids, homeologous genes from different sub‐genomes can exhibit very high sequence similarity, especially in coding regions, making it difficult to design genome‐specific primers that amplify individual gene copies (X. Q. Huang & Brûlé‐Babel, 2010). Moreover, cDNA rather than genomic DNA was used for RT‐PCR validation because the aim was not only to confirm the presence of candidate genomic regions but also to assess whether PAV predictions could be validated at the transcript level. However, this approach requires that the target gene display the expected expression pattern in the sampled tissue and developmental stage, thereby adding an additional constraint to candidate validation. Nevertheless, qualitative RT‐PCR analysis provided proof of principle that predicting genotype‐specific expression based on PAV is effective; in fact, qualitative RT‐PCR confirmed that the *PVA31*‐like gene was expressed exclusively in apomictic genotypes, as predicted by our PAV analysis. *PVA31*‐like is homologous to *A. thaliana PVA31*, a vesicle‐associated membrane protein involved in salicylic acid‐mediated programmed cell death (PCD) (Ichikawa et al., 2015). Since PCD is a hallmark of tissues from which apomictic embryos originate, it may also actively participate in plant embryogenesis (Pennell & Lamb, 1997). Therefore, *PVA31*‐like may link intracellular trafficking, cell death‐related signaling, and reproductive cell fate decisions. Interestingly, another *PVA* gene homologous to *A. thaliana PVA12* was previously reported to be absent in the sexual diploid cv. Victoria and present in apomictic cytotypes of *E. curvula* (Carballo et al., 2023). In *A. thaliana*, *PVA12* interacts with a DC1 domain‐containing protein required for vacuole formation in male and female gametophytes; without it, development arrests before the first post‐meiotic mitotic division (D'Ippólito et al., 2017). Together, these observations suggest that vesicle‐associated proteins may contribute to apomictic development in *E. curvula*.

In addition, the in silico screening of primer pairs previously developed to amplify the *EcAPO* candidate genes enabled comparison of these previously proposed candidates with the PAV‐derived gene sets. In our pangenome, the targets of the *EcAPO1*‐ and *EcAPO2*‐specific primers were recovered among the apomixis‐associated genes, whereas those of the *EcAPO3*‐ and *EcAPO4*‐specific primers were identified among the polyploid‐associated genes. The corresponding pangenome gene models were annotated as a hypothetical protein, a *CWC22*‐like pre‐mRNA splicing factor, an A‐type cyclin, and an F‐box protein, respectively, consistent with both the protein annotations previously reported for these *EcAPO* candidates and their distribution across *E. curvula* cytotypes (Garbus et al., 2026). The recovery of two previously characterized *EcAPO* primer targets within each PAV‐derived gene set shows consistency between the previously proposed candidates and our PAV‐derived classification.

Regarding reproduction in *E. curvula*, the near absence of pangenome‐variable genes unique to sexual accessions suggests that apomictic cytotypes retain most of the sexual gene pool while carrying additional genes absent in sexual accessions. This supports the idea that apomixis should not be regarded merely as a loss of sexuality but rather as an alternative reproductive strategy that may be anciently polyphenic with sex and involve canalized components of complex molecular processes (Albertini et al., 2019). Epigenetic regulation and small RNA pathways are believed to mediate the switch between sexuality and apomixis (Garbus et al., 2019). Consistent with this, a kinesin gene has been reported to be repressed in the apomictic Tanganyika cultivar via miRNA (microRNA)‐mediated regulation (Pasten et al., 2022), and increased DNA methylation has been observed in apomictic genotypes compared to sexual ones (Carballo, Zappacosta, Marconi, et al., 2021). Under stressful conditions such as drought or tissue culture, the percentage of sexual embryo sacs increases (Rodrigo et al., 2017; Selva et al., 2020), providing evidence not only of a possible epigenetic mechanism involved in this shift but also that apomictic plants retain, at least partially, the capacity for sexual reproduction. Functional enrichment analyses further support this idea. Polyploid‐associated genes were highly enriched in GO categories related to cell‐cycle regulation and DNA replication, as well as in terms associated with developmental, structural, and transport processes (Figure 4a; Table S6). Among these genes, the strongest enrichment signal was derived from A‐type cyclin (*CYCA*) homologs, a class of *CDK* regulators that plays a crucial role in cell‐cycle control (Renaudin et al., 1996). Based on sequence homology, plant cyclins have been classified into nine groups (De Veylder et al., 1999; Renaudin et al., 1996), with the A‐type cyclins (*CYCAs*) comprising the A1, A2, and A3 groups. Some members of the *CYCA* class are active during S‐phase progression (De Veylder et al., 1999), when the genome is duplicated, and two sister chromatids are generated for each chromosome. Given their role during DNA replication and, more broadly, during cell‐cycle regulation, alterations affecting *CYCAs* could contribute to cellular polyploidization. Therefore, the enrichment of A‐type cyclins among the polyploid‐associated gene set is especially notable. *In Arabidopsis*, specific A‐type group‐2 cyclins (*CYCA2s*) have been directly linked to ploidy and reproductive development. *CYCA2;3* controls ploidy levels during endoreduplication (Imai et al., 2006), whereas *CYCA2;1* is expressed in the ovule apparatus before fertilization and during embryogenesis in both the embryo and the endosperm (Burssens et al., 2000). Additionally, the *Arabidopsis* genes *CYCA1;2*, an A‐type group‐1 cyclin also known as TARDY ASYNCHRONOUS MEIOSIS (*TAM*) (d'Erfurth et al., 2010; Y. Wang et al., 2004), and *OSD1* (d'Erfurth et al., 2009) are essential for meiotic cell‐cycle progression, and mutations in these genes can result in the formation of viable unreduced gametes (Brownfield & Kohler, 2011). Notably, *EcAPO3*, a homolog of the *Oryza brachyantha* Cyclin‐2A‐1 protein, was recently proposed as a potential regulator of apomixis in *E. curvula* (Garbus et al., 2026). Using a pangenomic approach, we identified three cyclin type‐A gene models among the polyploid‐associated genes that contributed to the GO enrichment signal (Table S8). These gene models were present in all polyploid cytotypes and absent from all diploid cytotypes (Table S10). In silico screening with the *EcAPO* primers showed that one of these gene models corresponded to *EcAPO3*, which was annotated as a *Cyclin A2;1*‐like gene based on the pangenome's best‐hit annotation (Table S7). Together, these results support the association between cyclin type‐A genes and polyploid cytotypes.

Additional enriched categories among polyploid‐associated genes were supported by genes annotated as kinesin homologs, including members of the *KIN‐7* subfamily. In *A. thaliana*, *KIN‐7* (also known as *KAK1*/*POS3*) is involved in chromosomal congression and kinetochore‐microtubule attachment. Its loss causes defects in chromosomal alignment and delays the onset of anaphase, consistent with a role in mitotic progression and chromosomal segregation (Kriechbaum & Müller, 2026). Interestingly, a kinesin‐like protein (*KIN‐14D*) had previously been reported to be repressed by a miRNA in an apomictic cultivar (i.e., Tanganyika INTA) of *E. curvula*, suggesting a potential role in the expression of the reproductive mode (Pasten et al., 2022).

In contrast, genes associated with apomixis were mainly enriched in RNA processing and post‐transcriptional regulation (Figure 4b; Table S8). The strongest enrichment signal was linked to a homolog of the spliceosomal factor *CWC22*, a conserved component essential for the deposition of the exon junction complex and efficient pre‐mRNA splicing (Steckelberg et al., 2012). Functional studies in other organisms suggest that *CWC22* may play important roles at the cellular level. In humans, *CWC22* knockdown causes mitotic slippage, leading to premature mitotic exit due to impaired spindle assembly checkpoint function after prolonged prometaphase (Yuki et al., 2026). In the housefly, male sex determination is controlled by *Mdmd*, a paralog of the general *CWC22* splicing factor gene (Sharma et al., 2017). *Mdmd* is found only in the housefly, and *CWC22* is not known to play a role in sex determination in other insects, highlighting the potential for de novo recruitment of paralogs of unrelated genes into the sex‐determination pathway (Vicoso, 2019). In mice, increasing the copy number of *CWC22* at the *R2d2* locus results in distortion of female meiotic drive and maternal transmission ratio (Didion et al., 2015).

Although such mechanisms have not yet been demonstrated in plants, these observations suggest that variation among *CWC22*‐like gene models, potentially involving copy number, sequence divergence, or expression, could affect meiotic regulation or post‐transcriptional control of reproductive development. In this context, regulation of RNA splicing may serve as an additional layer of control, helping to establish or stabilize the apomictic pathway. Supporting this idea, distinct *CWC22*‐like gene models were identified among the variable genes in the pangenome. Four of these were present in all apomictic accessions and absent in all sexual cytotypes, while one additional *CWC22*‐like gene model was found only in the tetraploid Tanganyika USDA and the hexaploid Don Luis (Table S10).

The recovery of *EcAPO1* and *EcAPO4* in our PAV‐derived gene sets suggests that candidate genes that do not directly contribute to significantly enriched GO categories may still be biologically relevant. For example, the 582 polyploid‐associated gene set (Table S7) also included a *CDKG1*‐like gene (Table S10). In *A. thaliana*, *CDKG1* regulates pre‐mRNA splicing of *CALLOSE SYNTHASE5* (*CalS5*), thereby affecting pollen wall formation (X.‐Y. Huang et al., 2013). Moreover, *CDKG1* is differentially spliced under low‐temperature and ambient‐temperature variation, and its splice variants have been implicated in male meiosis and synapsis (Verhage et al., 2017). Although the *CDKG1*‐like gene did not directly contribute to the enriched GO categories discussed above, its presence in the polyploid‐associated set is noteworthy. *CDKG* genes belong to a clade of cyclin‐dependent protein kinases related to *CDK10*/*CDK11* and to *Ph1*‐related kinases, which have been implicated in the control of homoeologous chromosome pairing in wheat (Verhage et al., 2017). Therefore, the *CDKG1*‐like candidate identified here may be primarily relevant to the polyploid‐associated meiotic regulation, with possible downstream effects on reproductive development.

Beyond the GO enrichment results, the analysis of the PAV‐derived gene sets also highlighted regulatory components that may help distinguish polyploidy‐ and apomixis‐associated candidates. Analysis of TF/TR families, which are important players in apomixis and polyploidy (Palumbo et al., 2022; Springer & Musiał, 2026), showed that the candidate gene sets include both shared and category‐specific regulatory families. FAR1, B3, and TRAF families were detected in both gene sets, suggesting that these regulatory families are not exclusive to either polyploidy‐ or apomixis‐associated genes. The recurrent detection of FAR1‐related genes may reflect the broad functional range of this family, which has been implicated in light signaling and several developmental processes (Ma & Li, 2018). B3 TFs include embryogenesis‐related regulators such as LEC2 and FUS3 (Stone et al., 2008; Wu et al., 2020), whereas TRAF‐related regulators have been associated with megaspore mother cell specification and female gametophyte development (Singh et al., 2017). In contrast, AP2/ERF TFs were detected only among apomixis‐associated genes. This is noteworthy because this family includes BABY BOOM/PLETHORA‐like regulators involved in embryogenesis (Chen et al., 2022), and it has been shown that ectopic expression of BABY BOOM1 in the egg cell is sufficient for parthenogenesis in rice (Khanday et al., 2019). Together, these observations indicate that FAR1, B3, and TRAF are shared regulatory families across the two gene sets, whereas other TF families show category‐specific distributions. DOF, LOB/LBD, PLATZ, ZF‐HD, and C2H2 were detected among polyploid‐associated genes, while AP2/ERF, MYB, and NAC were detected among apomixis‐associated genes.

Taken together, these results indicate that polyploidy and apomixis in *E. curvula* are associated with two gene groups. GO enrichment analyses highlighted distinct functional trends between them: polyploid‐associated genes mainly related to cell‐cycle regulation, DNA replication, and chromosome‐associated processes, and apomixis‐associated genes mainly related to RNA processing and spliceosome‐related functions. However, additional genes that do not directly contribute to enriched GO categories, including *EcAPO1*, *EcAPO4*, and the *CDKG1*‐like gene, were also recovered within the PAV‐derived gene sets. Further analyses will be needed to clarify the specific functions of these genes and to determine whether interactions between polyploidy‐ and apomixis‐associated gene groups may contribute to the frequent association between polyploidy and apomixis observed in this species.

Our SNP‐based analysis confirmed the previously recognized historical and molecular relationships among the cytotypes studied. Tanganyika INTA and Bahiense clustered together, indicating a shared breeding history (Cardone et al., 2006), while Ermelo showed substantial similarity to this group. Victoria clustered with PI299920, whereas PI208214 was positioned close to OTA‐S, consistent with previous simple sequence repeat based findings (Carballo et al., 2019). However, the greater similarity among Tanganyika INTA, Bahiense, and Ermelo, which form a subcluster distinct from the diploids Victoria and PI299920, may indicate hybridization involving Ermelo in the breeding history of Bahiense and Tanganyika INTA. OTA‐S, the result of USDA breeding activities using South African *E. curvula* germplasm (Voigt, 1976), was positioned close to PI208214, suggesting a shared genetic background between these two accessions. In contrast, Tanganyika USDA formed a separate branch, which may be related to its fully apomictic reproductive mode. Notably, apomictic cytotypes were distributed across different branches of the network rather than forming a single cluster, suggesting that genome‐wide divergence is not explained by reproductive mode alone. The high‐polyploid accessions, including Don Luis, Don Pablo, and TUNS9355, clustered close to Don Eduardo, a pattern that could be associated with their Argentine origin (Luciani et al., 2012).

SNP markers revealed ploidy‐associated patterns across *E. curvula* cytotypes when analyzed using both SNP‐call heterozygosity and AF distributions. SNP‐call heterozygosity increased with ploidy, particularly in high‐polyploid facultative apomictic accessions. Although sequencing errors and read mismapping may inflate heterozygous SNP calls (Phillips, 2024), higher levels of allelic variation may also be expected in poorly domesticated polyploid genotypes, such as those analyzed here. Accordingly, when heterozygous SNPs were used to examine AF distributions, the observed peaks were compared with the expected AF values for each ploidy level rather than being used to infer allelic dosage at individual loci. In polyploid genomes, AF profiles may be influenced by uncertainties in read mapping, variant identification, genotype estimation, allelic dosage, sub‐genome homology, and chromosome inheritance patterns (Phillips, 2024). Nevertheless, allele‐balance information, which reflects read‐based allele frequencies at heterozygous sites, can reveal departures from a diploid state even from low‐coverage sequencing data (Fletcher et al., 2022). Consistent with these limitations, our results showed well‐defined AF peaks in diploid accessions and recognizable patterns in tetraploids, whereas the profiles of the higher ploidy accessions were progressively more difficult to interpret (Figure S8). As ploidy increases, the expected AF classes become more closely spaced, and their resolution may be further reduced by sequencing depth, the presence of multiple sub‐genomes in allopolyploids, and the possible segmental nature of chromosome inheritance, making individual AF peaks increasingly difficult to resolve.

In our dataset, polyploid accessions were sequenced to higher raw read depths to achieve genome‐wide coverage equal to or greater than that of diploid accessions (Table S1), thereby requiring proportionally greater sequencing effort due to their larger genome sizes. To assess whether differences among tetraploid AF profiles were mainly due to sequencing depth, Tanganyika INTA was included in the downstream analysis as a high‐coverage tetraploid accession, and SNP‐level mean depth was calculated for the heterozygous sites used in the AF analysis (Table S16). Bahiense, despite having a lower SNP‐level mean depth than Tanganyika INTA, showed a well‐resolved AF profile with three peaks at about 0.25, 0.5, and 0.75, highly similar to that of Tanganyika INTA. Given the reported origin of Bahiense through in vitro genome doubling (Cardone et al., 2006) and the autotetraploid‐like AF profile previously observed for Tanganyika INTA using a different reference genome (Carballo et al., 2023), our results are consistent with an autotetraploid‐like AF pattern in both accessions and suggest that the sequencing depth achieved for Bahiense was sufficient to resolve the main AF peaks in the present pangenome‐based analysis. By contrast, Tanganyika USDA showed a clearly dominant AF peak around 0.5, despite having SNP‐level mean depth comparable to those of OTA‐S and Ermelo (Table S16), a pattern consistent with an allotetraploid‐like AF profile. Therefore, the less sharply resolved profiles observed in OTA‐S and Ermelo do not appear to reflect sequencing depth differences alone and may instead be consistent with more complex polyploid behavior, potentially including segmental‐like patterns, although this cannot be resolved from AF data alone. This interpretation is also consistent with previous cytogenetic evidence suggesting segmental allopolyploidy and gene flow among morphological varieties of *E. curvula* (Poverene, 1988), whereas Tanganyika INTA showed a pattern consistent with autopolyploidy (Zeid et al., 2011). Overall, these results suggest that tetraploid cytotypes of *E. curvula* may not all share the same genomic behavior or evolutionary history and support the view that the distinction between autopolyploid and allopolyploid forms may represent a continuum rather than two discrete states (Comai, 2005). Diploid accessions showed AF profiles centered around 0.5, whereas tetraploid cytotypes displayed different combinations of peaks at about 0.25, 0.5, and 0.75, with differences in peak resolution and relative height. At higher ploidy levels, AF distributions were more heterogeneous and multi‐peaked, corresponding to the broader range of expected AF values in 6x–7x cytotypes, although these profiles remain difficult to interpret unambiguously.

## 5 CONCLUSIONS

In conclusion, we generated a linear pangenome of *E. curvula* that includes accessions with different ploidy levels and reproductive modes. This resource revealed extensive gene‐content variation and enabled the identification of PAV‐derived candidate gene sets associated with polyploidy and apomictic reproduction. GO enrichment analyses highlighted distinct functional signatures, with polyploid‐associated genes enriched for cell‐cycle regulation, DNA replication, and kinetochore‐ and spindle‐associated functions, whereas apomixis‐associated genes were enriched for RNA processing and spliceosome‐related functions. TF/TR analyses further revealed both shared and category‐specific regulatory families associated with polyploidy and apomixis. The identification of previously proposed *EcAPO* candidates within polyploid‐ or apomixis‐associated gene sets, together with additional candidates such as *PVA31*‐like, *CDKG1*‐like, *KIN‐7*‐like, *CWC22*‐like, and *cyclin*‐like genes, supports the relevance of the PAV‐derived candidates identified here. Shared and category‐specific TF/TR families further indicate that polyploidy and apomixis are associated with both common and distinct regulatory components. Together, PAV and SNP analyses highlight the mosaic nature of genome evolution in the E. curvula agamic complex, suggesting that polyploidization, genome restructuring, and shifts in reproductive mode may have contributed to the emergence of distinct genomic architectures. Overall, our data suggest that the frequent association between polyploidy and apomixis in this species may involve the combined contribution of two distinct groups of candidate genes, one associated with polyploidy and the other with apomictic reproduction, providing a basis for future functional validation in this taxonomically complex grass species. In addition, SNP and AF patterns revealed cytotype‐specific genomic differences, supporting future investigations of the possible auto‐, allo‐, or segmental‐like nature of polyploid genome organization through chromosome‐pairing analyses and/or synteny‐based comparisons.

## AUTHOR CONTRIBUTIONS

**Giacomo Bongiorno**: Conceptualization; data curation; formal analysis; methodology; writing—original draft. **José Carballo**: Conceptualization; data curation; formal analysis; methodology; writing—original draft. **Juan Pablo Selva**: Investigation. **Diego Zappacosta**: Investigation. **Emidio Albertini**: Conceptualization; funding acquisition; writing—original draft. **Viviana Echenique**: Conceptualization; funding acquisition; writing—original draft.

## CONFLICT OF INTEREST STATEMENT

The authors declare no conflicts of interest.

## Supporting information




**Supplemental Table S1**. Sequencing data and metadata summary. The table reports accession panel numbering, pangenome assembly order, available and newly generated genomic resources, sequencing files, read statistics, quality metrics, GC content, total sequenced bases, and genome coverage. Coverage (x) indicates estimated sequencing depth, whereas Effective (%) indicates the proportion of high‐quality bases retained after filtering. (C) denotes accessions collected from natural populations, with the corresponding collection site indicated, (D) denotes cultivars developed through breeding programs, and (G) and (U) indicate resources generated and used in this study, respectively. Victoria was the initial reference genome, accessions (assembly order 1–10) were sequentially added to the pangenome, and Tanganyika INTA was used only for downstream analyses.
**Supplemental Table S2**. Oligonucleotide primers used for PAV validation and in silico analyses. The table lists the names, sequences, and sources of the gene‐specific primers designed in this study for qualitative RT‐PCR validation of selected PAV‐derived candidate genes, the UBICE primers previously developed by Garbus et al. (2017) and used as an internal amplification control, and the *EcAPO* primer pairs previously developed by Garbus et al. (2026) and used for in silico PCR analysis.
**Supplemental Table S3**. Assembly metrics for each *Eragrostis curvula* accession included in the iterative pangenome construction. The table reports the ploidy level, reproductive mode, iteration stage, number of contigs/scaffolds added at each iteration and cumulatively, number of bases added to the pangenome, cumulative pangenome size, N50, and GC content of the sequences added at each iteration.
**Supplemental Table S4**. Summary statistics of the pan‐genome assembly and annotation. The table reports assembly length before and after filtering, the number of bases removed during filtering, N50, GC content, number of predicted genes, proportion of repetitive elements, and BUSCO completeness scores for the final linear pan‐genome assembly.
**Supplemental Table S5**. Gene presence/absence variation and statistical comparison of gene counts among *Eragrostis curvula* accessions. Panel A reports the ploidy level and the numbers of genes classified as absent or present for each accession based on PAV analysis of the final pangenome. Panel B summarizes the number of present genes by ploidy level. Panel C reports the results of Welch's t‐tests comparing gene counts among ploidy groups and between diploid and polyploid accessions, as well as the linear regression analysis relating gene number to ploidy level.
**Supplemental Table S6**. Gene sets associated with polyploidy and apomixis identified by PAV analysis. The table reports the 582 genes absent from all diploid accessions and present in all polyploid accessions, and the 406 genes present in all apomictic accessions and absent from all sexual accessions.
**Supplemental Table S7**. In silico PCR analysis of *EcAPO* primer pairs against the *Eragrostis curvula* pangenome. The table reports the *EcAPO* primer pairs previously described by Garbus et al. (2026), the pangenome scaffolds yielding predicted amplicons, the orientation, coordinates, and length of each amplicon, the overlapping gene models, their PAV distribution across accessions, and functional annotations obtained using the NCBI nr plant protein database (PLANT_DB), the *Oryza sativa* STRING v12 protein dataset (O. sativa), and the *Arabidopsis thaliana* STRING v12 protein dataset (A. thaliana).
**Supplemental Table S8**. Gene Ontology enrichment results for polyploid‐associated genes. The table reports the significantly enriched Gene Ontology terms identified for the 582 polyploid‐associated genes, including the GO identifier, term description, raw P value, adjusted P value, q value, number of genes associated with each term, corresponding gene model identifiers, and functional annotations obtained using the NCBI nr plant protein database (PLANT_DB), the *Oryza sativa* STRING v12 protein dataset (O. sativa), and the *Arabidopsis thaliana* STRING v12 protein dataset (A. thaliana).
**Supplemental Table S9**. Gene Ontology enrichment results for apomixis‐associated genes. The table reports the significantly enriched Gene Ontology terms identified for the 406 apomixis‐associated genes, including the GO identifier, term description, raw P value, adjusted P value, q value, number of genes associated with each term, corresponding gene model identifiers, and functional annotations obtained using the NCBI nr plant protein database (PLANT_DB), the *Oryza sativa* STRING v12 protein dataset (O. sativa), and the *Arabidopsis thaliana* STRING v12 protein dataset (A. thaliana).
**Supplemental Table S10**. Distribution across accessions and functional annotation of selected gene models. The table includes three A‐type Cyclin‐like genes and four *CWC22*‐like genes associated with significantly enriched Gene Ontology terms, a *CDKG1*‐like gene included among the 582 polyploid‐associated genes, and an additional *CWC22*‐like gene detected only in Tanganyika USDA and Don Luis. For each gene model, the accessions in which it was present or absent and the functional annotations obtained using the NCBI nr plant protein database (PLANT_DB), the *Oryza sativa* STRING v12 protein dataset (O. sativa), and the *Arabidopsis thaliana* STRING v12 protein dataset (A. thaliana).
**Supplemental Table S11**. Transcription factor and transcriptional regulator identified in polyploids. The table reports the Gene ID, Family Name, regulatory Category (TF or TR), and detailed family assignment (Detail) for TFs and TRs identified by iTAK.
**Supplemental Table S12**. Transcription factor and transcriptional regulator identified in apomictics. The table reports the Gene ID, Family Name, regulatory Category (TF or TR), and detailed family assignment (Detail) for TFs and TRs identified by iTAK.
**Supplemental Table S13**. Summary statistics for the filtering steps used to generate the SNP dataset for phylogenetic analysis. The table reports the number of samples and variant records retained at each step, including SNP retention and InDel removal, selection of biallelic SNPs, retention of SNPs with no more than three missing sites, selection of neutral and intergenic sites, and filtering of linked SNPs to retain independent sites.
**Supplemental Table S14**. Variant‐calling and filtering statistics for each accession. The table reports the ploidy level and reproductive mode of each accession, together with the number of retained sites, mean sequencing depth, and fraction of missing genotypes calculated after each filtering step: SNP retention and InDel removal, selection of biallelic SNPs, retention of biallelic SNPs with fewer than three missing genotypes, selection of neutral and intergenic SNPs, and filtering of linked SNPs to retain independent sites. Missing‐genotype fractions are reported as proportions (0‐1).
**Supplemental Table S15**. Genome‐wide SNP genotype statistics per accession. The table reports, for each accession, the ploidy level, reproductive mode, total number of non‐missing SNP genotype calls, and the numbers of heterozygous, homozygous reference, and homozygous alternative genotype calls. The corresponding fractions are also reported. Total homozygous fractions were calculated as the sum of homozygous reference and homozygous alternative fractions. Fraction values are reported as proportions (0‐1).
**Supplemental Table S16**. Mean sequencing depth at heterozygous SNP sites used for allele‐frequency (AF) analysis. The table reports, for each accession, the ploidy level, reproductive mode, the number of heterozygous SNP sites included in the AF analysis, and the corresponding mean sequencing depth. Mean depth values are reported as average read depth (x).

**Supplemental Figure S1**. Total sequence length contributed by each *Eragrostis curvula* accession during iterative pangenome assembly. Colors indicate ploidy levels: 2x (light blue), 4x (red), 6x (yellow), and 7x (purple).
**Supplemental Figure S2**. Cumulative pangenome growth curve of *Eragrostis curvula*. Each point represents the cumulative length of the pangenome after the incorporation of novel sequences contributed by each accession in the order indicated on the x‐axis. The orange line corresponds to the genome size of the *E. curvula* cv. Victoria reference (603 Mb), while the blue line shows the cumulative increase in assembly size up to ∼899 Mb after the last iteration.
**Supplemental Figure S3**. Heatmap of gene presence/absence variation (PAV) across *Eragrostis curvula* accessions. It shows the distribution of present (green) and absent (red) genes among *Eragrostis curvula* accessions.
**Supplemental Figure S4**. RT‐PCR amplification of *PVA31*‐like and *UBICE* using cDNA from *Eragrostis curvula* spikelets. Gel electrophoresis shows successful amplification of *PVA31*‐like (a) exclusively in apomictic accessions, while *UBICE* (b), used as a positive control, was successfully amplified in all accessions.
**Supplemental Figure S5**. Distribution of transcription factor and transcriptional regulator families in polyploid‐ and apomictic‐associated genes. Each bar represents the number of genes assigned to each TF/TR family based on conserved protein domains. Counts are shown separately for the polyploid‐associated (green) and apomictic‐associated (blue) genes.
**Supplemental Figure S6**. Principal component analysis (PCA) based on 1,696,724 neutral, intergenic, unlinked SNPs. PC1 and PC2 explained 23.6% and 20.6% of the total genetic variation, respectively. Colors indicate ploidy level (2x, light blue; 4x, red; 6x, yellow; 7x, purple), and symbols indicate reproductive mode (circles, sexual; squares, apomictic).
**Supplemental Figure S7**. Proportion of homozygous and heterozygous SNP loci across *Eragrostis curvula* accessions. Diploid sexual accessions show high homozygosity, tetraploids display intermediate profiles, and high‐polyploid apomictic accessions exhibit the highest heterozygosity.
**Supplemental Figure S8**. Genome‐wide allele‐frequency (AF) distributions across *Eragrostis curvula* accessions. Histograms show allele‐frequency values on the x‐axis and heterozygous SNP counts (×1000) on the y‐axis. Vertical dashed lines indicate the expected AF values for different ploidy levels, including 1/6, 1/4, 1/3, 1/2, 2/3, 3/4, and 5/6. Panels correspond to: (a) PI208214, (b) PI299920, (c) Tanganyika INTA, (d) Tanganyika USDA, (e) Bahiense, (f) OTA‐S, (g) Ermelo, (h) Don Luis, (i) TUNS9355, (j) Don Eduardo, and (k) Don Pablo.

## Data Availability

Raw sequencing reads have been deposited in the NCBI Sequence Read Archive under BioProject PRJNA1321158. The pangenome assembly and annotation are available in Zenodo at https://www.ncbi.nlm.nih.gov/sra/?term=PRJNA1321158.
