# Supplementary material for: A pangenome‐based approach reveals genes associated with polyploidy and apomixis in Eragrostis curvula
Source: Plant Genome. 2026 Sep 21;19(3):e70303. doi: 10.1002/tpg2.70303 (PMC13591433; doi:10.1002/tpg2.70303)
Supplement: Supplementary file 1 [file TPG2-19-e70303-s002.docx]

**Authors:** Giacomo Bongiorno, José Carballo, Juan Pablo Selva, Diego Zappacosta, Emidio Albertini and Viviana Echenique.

**Manuscript title:** A Pangenome-Based Approach Reveals Genes Associated with Polyploidy and Reproductive Mode in *Eragrostis curvula*

**Supplemental Figure S1.** Total sequence length contributed by each *Eragrostis curvula* accession during iterative pangenome assembly. Colors indicate ploidy levels: 2x (light blue), 4x (red), 6x (yellow), and 7x (purple).

**Authors:** Giacomo Bongiorno, José Carballo, Juan Pablo Selva, Diego Zappacosta, Emidio Albertini and Viviana Echenique.

**Manuscript title:** A Pangenome-Based Approach Reveals Genes Associated with Polyploidy and Reproductive Mode in *Eragrostis curvula*

**Supplemental Figure S2**. Cumulative pangenome growth curve of *Eragrostis curvula*. Each point represents the cumulative length of the pangenome after the incorporation of novel sequences contributed by each accession in the order indicated on the x-axis. The orange line corresponds to the genome size of the *E. curvula* cv. Victoria reference (603 Mb), while the blue line shows the cumulative increase in assembly size up to ~899 Mb after the last iteration

.

**Authors:** Giacomo Bongiorno, José Carballo, Juan Pablo Selva, Diego Zappacosta, Emidio Albertini and Viviana Echenique.

**Manuscript title:** A Pangenome-Based Approach Reveals Genes Associated with Polyploidy and Reproductive Mode in *Eragrostis curvula*

**Supplemental Figure S3**. Heatmap of gene presence/absence variation (PAV) across *Eragrostis curvula* accessions. It shows the distribution of core (green) and variable (red) genes among *Eragrostis curvula* accessions.

**Authors:** Giacomo Bongiorno, José Carballo, Juan Pablo Selva, Diego Zappacosta, Emidio Albertini and Viviana Echenique.

**Manuscript title:** A Pangenome-Based Approach Reveals Genes Associated with Polyploidy and Reproductive Mode in *Eragrostis curvula*

**Supplemental Figure S4**. RT-PCR amplification of *PVA31*-like and *UBICE* using cDNA from *Eragrostis curvula* spikelets. Gel electrophoresis shows successful amplification of *PVA31*-like (a) exclusively in apomictic accessions, while *UBICE* (b), used as a positive control, was successfully amplified in all accessions.

**Authors:** Giacomo Bongiorno, José Carballo, Juan Pablo Selva, Diego Zappacosta, Emidio Albertini and Viviana Echenique.

**Manuscript title:** A Pangenome-Based Approach Reveals Genes Associated with Polyploidy and Reproductive Mode in *Eragrostis curvula*

**Supplemental Figure S5**. Distribution of transcription factor and transcriptional regulator families in polyploid- and apomictic-associated genes. Each bar represents the number of genes assigned to each TF/TR family based on conserved protein domains. Counts are shown separately for the polyploid-associated (green) and apomictic-associated (blue) genes.

**Authors:** Giacomo Bongiorno, José Carballo, Juan Pablo Selva, Diego Zappacosta, Emidio Albertini and Viviana Echenique.

**Manuscript title:** A Pangenome-Based Approach Reveals Genes Associated with Polyploidy and Reproductive Mode in *Eragrostis curvula*

**Supplemental Figure S6**. Principal component analysis (PCA) based on 1,696,724 neutral, intergenic, unlinked SNPs. PC1 and PC2 explained 23.6% and 20.6% of the total genetic variation, respectively. Colors indicate ploidy level (2x, light blue; 4x, red; 6x, yellow; 7x, purple), and symbols indicate reproductive mode (circles, sexual; squares, apomictic).

**Authors:** Giacomo Bongiorno, José Carballo, Juan Pablo Selva, Diego Zappacosta, Emidio Albertini and Viviana Echenique.

**Manuscript title:** A Pangenome-Based Approach Reveals Genes Associated with Polyploidy and Reproductive Mode in *Eragrostis curvula*

**Supplemental Figure S7**. Proportion of homozygous and heterozygous SNP loci across *Eragrostis curvula* accessions. Diploid sexual accessions show high homozygosity, tetraploids display intermediate profiles, and high-polyploid apomictic accessions exhibit the highest heterozygosity.

**Authors:** Giacomo Bongiorno, José Carballo, Juan Pablo Selva, Diego Zappacosta, Emidio Albertini and Viviana Echenique.

**Manuscript title:** A Pangenome-Based Approach Reveals Genes Associated with Polyploidy and Reproductive Mode in *Eragrostis curvula*

**Supplemental Figure S8**. Genome-wide allele-frequency (AF) distributions across *Eragrostis curvula* accessions. Histograms show allele-frequency values on the x-axis and heterozygous SNP counts (×1000) on the y-axis. Vertical dashed lines indicate the expected AF values for different ploidy levels, including 1/6, 1/4, 1/3, 1/2, 2/3, 3/4, and 5/6. Panels correspond to: (a) PI208214, (b) PI299920, (c) Tanganyika INTA, (d) Tanganyika USDA, (e) Bahiense, (f) OTA-S, (g) Ermelo, (h) Don Luis, (i) TUNS9355, (j) Don Eduardo, and (k) Don Pablo.
